# Level and determinants of district primary healthcare system technical efficiency in Ghana: two-stage stochastic frontier analysis

**DOI:** 10.1136/bmjgh-2024-018847

**Published:** 2026-02-12

**Authors:** Beatrice Amboko, Jacob Novignon, Rose Nabi Deborah Karimi Muthuri, Fiammetta Maria Bozzani, Anna Vassall, Edwine Barasa

**Affiliations:** 1 Health Economics Research Unit, KEMRI-Wellcome Trust Research Programme, Nairobi, Kenya; 2 Nuffield Department of Medicine, University of Oxford, Oxford, UK; 3 Centre for Social Policy Studies, University of Ghana, Accra, Ghana; 4 Department of Global Health and Development, London School of Hygiene & Tropical Medicine, London, England, UK; 5 Global Health and Development, London School of Hygiene & Tropical Medicine, London, England, UK; 6 Amsterdam Institute of Global Health and Development, Academic Medical Centre, Amsterdam, Netherlands

**Keywords:** Global Health, Health systems, Health economics, Public Health

## Abstract

**Background:**

Primary healthcare (PHC) is critical towards achieving Universal Health Coverage (UHC). In Ghana, PHC is organised at the district level and plays a key role in the country’s pursuit of UHC. However, many districts face challenges not only with limited resources but also with how effectively they are used. We examined how efficiently districts in Ghana use their health resources and what factors are associated with this efficiency.

**Methods:**

We used a two-step stochastic frontier analysis model using data from 181 districts. The output variable was a composite coverage index derived from eight PHC service indicators for 2021, primarily reflecting maternal and child health and infectious disease services. Input variables included district health expenditure for 2020/2021 and the number of health facilities and clinical staff in 2021. We then assessed the associations between efficiency scores generated by the model and health systems, socioeconomic and demographic factors, such as health facility type, insurance coverage, literacy level, Gini coefficient, poverty incidence, urbanisation and population density.

**Results:**

On average, districts operated at 87% efficiency, with scores ranging from 65% to 99%. Two factors were associated with the efficiency. First, districts with a higher proportion of PHC facilities tended to use resources more efficiently (coeff=0.151; 95% CI=0.041 to 0.261). Second, districts with greater income inequality were less efficient, measured by the Gini coefficient (coeff=−0.858; 95% CI=−1.146 to −0.252).

**Conclusion:**

Districts in Ghana have the potential to improve PHC outputs by about 13% on average by better use of existing resources and addressing determinants of efficiency. Findings suggest that districts with a higher proportion of PHC facilities and lower income inequality tend to be more efficient. These patterns highlight the value of strengthening PHC infrastructure and pursuing equity-focused policies as part of strategies to enhance efficiency in district health systems.

**WHAT IS ALREADY KNOWN ON THIS TOPIC**

- Primary healthcare (PHC) systems in low- and middle-income countries often struggle with inadequate resources and inefficient use of the available resources.
- Assessing efficiency and its drivers is essential for improving PHC performance.
- Previous studies have assessed PHC efficiency with emphasis on specific health facilities to the neglect of the district health system as a unit.

**WHAT THIS STUDY ADDS**

- PHC efficiency in Ghana varies widely across districts, based on maternal, newborn and child health (MNCH) and infectious disease service delivery.
- Efficiency is associated with the proportion of PHC facilities and levels of income inequality.

**HOW THIS STUDY MIGHT AFFECT RESEARCH, PRACTICE, OR POLICY**

- Districts with more PHC facilities and lower income inequality tended to be more efficient, suggesting these may be areas for further exploration in efforts to improve efficiency.
- Findings can guide targeted interventions and inform policy reforms for optimising PHC service delivery.
- Identifying and addressing these systemic inefficiencies may create resource savings that are essential in the midst of growing resource limitations.

## Background

 Like many other low- and middle-income countries, Ghana has pledged to achieve Universal Health Coverage (UHC).^1^ In 2020, Ghana’s current spending on health was 4% of the country’s gross domestic product,^2^ which was below the 5% recommended level for achieving UHC. In line with Ghana’s push for UHC, the country has a decentralised governance system for health.^3^,^5^ Decentralisation has been endorsed as a fundamental reform for improving the provision of public health services.^6 7^ Ghana adopted a mixed type of administrative decentralisation of health governance that includes delegation, deconcentration and, more recently, devolution.^48^,^12^ The Ministry of Health (MoH) is the main governing body responsible for policy-making, national planning, coordination and overseeing teaching and specialised hospitals.^3 8^ The MoH delegated service delivery to a semi-autonomous agency, the Ghana Health Service (GHS), established in 1996 under the Ghana Health Service and Teaching Hospitals Act 525.^3^,^58 10 11 13 14^

**Figure 1:**
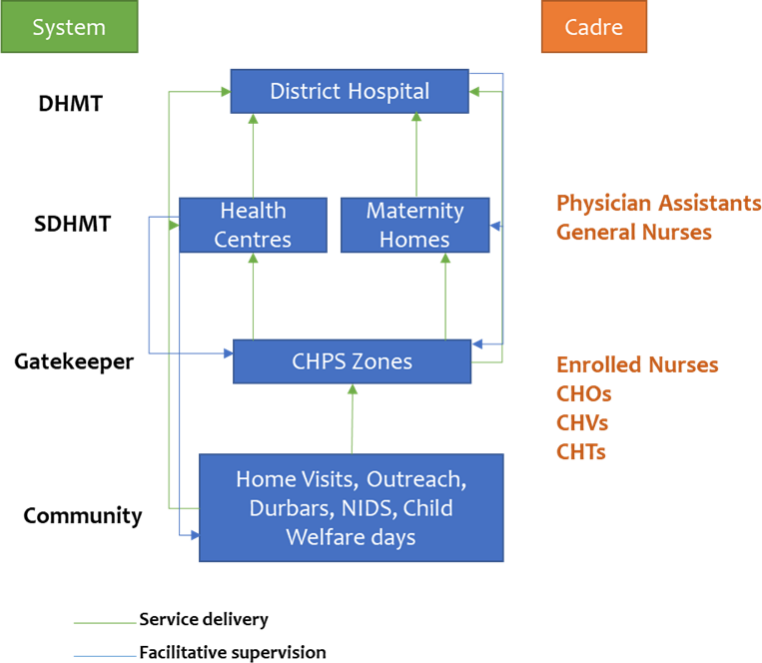
Ghana PHC system.^1^ Durbars are community gatherings used to share health information, promote education and engage the public in culturally significant ways. Abbreviations: SDHMT: Sub-District Health Management Team; CHOs: Community Health Officers; CHPS: Community-Based Health Planning and Services; CHTs: Community Health Teams; CHVs: Community Health Volunteers; DHMT: District Health Management Team; NIDS: National Immunisation Days.

Ghana has a three-tier health delivery system comprising district (primary), regional (secondary) and national (tertiary) levels. Primary healthcare (PHC) is delivered at the district level. The district health services are organised into three levels. The first level is the district level, which includes district hospitals that provide preventive, curative and promotive healthcare services. The second level comprises sub-district health centres, clinics and maternity homes, which serve as the first point of contact with the formal health system and provide basic preventive and curative services. The third and lowest level is the Community-based Health and Planning Services (CHPS), which provide preventive, promotive and treatment of common/minor illnesses (figure 1).^1^

Districts have their own District Assembly Common Funds that provide the necessary resources for meeting the development needs of their communities. However, more than half of district-level expenditures are executed at the central level, specifically for payment of staff and investments. The districts do not have absolute control over their spending, with centrally defined guidelines dictating some allocations. Moreover, some funds are earmarked for specific programmes. The districts rely on the government budget, financial credits, internally generated funds, national health insurance scheme reimbursements and donor-funded programmes.^5^

The performance of district health systems can, therefore, be attributed to their ability to efficiently and effectively allocate and use the available resources. Moreover, achieving UHC in Ghana will need additional funding, which can only be justified if the current resources are efficiently used.^15 16^ Improved health system efficiency has been reported to create fiscal space for health by freeing up or reallocating resources within existing budgets to increase investment in health.^15 17^ Efficiency is the degree to which a system meets its objectives given the invested resources. Technical efficiency is accomplished when the allocation of resources is such that outputs are maximised for a given level of inputs or inputs are minimised for a given level of output.^18^

Understanding health system efficiency and the factors associated with it is an important research and policy question, given the important role of efficiency in improving the use of available resources and unlocking additional resources. The technical efficiency of health systems has been examined across Africa.^19^,^22^ In Ghana, several studies have reported variations in efficiency and the associated factors at the health facility level.^23^,^28^ However, there is currently no evidence on the efficiency of the district PHC system or the factors associated with it. We aimed to address this critical research gap by estimating the technical efficiency of district PHC systems in Ghana, with a particular focus on PHC outputs. We also aimed to identify key determinants of efficiency at the district level. This innovative approach shifts the lens from individual health facilities to entire district health systems, key operational units within Ghana’s decentralised healthcare system. Examining the efficiency of the district health systems in Ghana is important due to their central role in providing PHC and significant consumption of resources. For instance, the district health system consumed 50% of the MoH spending in the fiscal year 2006–2008.^5^ In this study, we estimated the technical efficiency of district PHC systems in Ghana and its determinants using a proxy for PHC outputs based on a composite index that primarily reflects maternal, child health and infectious disease services.

## Methods

### Study design

This was a cross-sectional study using secondary data to parametrise a stochastic frontier analysis (SFA) model of PHC service provision at the district level in Ghana.

### Data sources

The data utilised for this study were obtained from the District Health Information Management System 2 (DHIMS2) for 2021, 2020/2021 budget allocation reports, 2021 population and housing census, and the poverty mapping report for 2015, as outlined in table 1. Data from 260 (95%) districts from 16 regions in Ghana was available. We included 181 (66%) districts in the efficiency analysis after dropping districts with missing clinical staff (3), budget (26), admissions (46), TB treatment (3) and HIV prevalence data (1) data. A total of 175 (64%) districts were included in the analysis of the effect of exogenous variables on efficiency due to missing data for poverty incidence and the Gini coefficient (6).

**Table 1:** Definition of input, output and exogenous variables

Variable	Description	Data source	Data transformation
Output variable			
Composite coverage index	A composite variable derived from eight output indicators. Percentage of women (15–49 years) that had family planning demand satisfied using modern methods Percentage of ANC clients who made the fourth visit Percentage of women who had skilled deliveries Measles-Rubella 2 vaccine coverage (ages 18–59 months) BCG vaccine coverage under 1 year Percentage of children that received DPT3 vaccine (under 1) Percentage of TB cases that completed treatment Percentage of ANC registrants that received LLINs	DHIMS2 for the year 2021	No transformation
Inputs			
Number of health facilities	Total number of health facilities per 10 000 population	DHIMS2 for the year 2021	No transformation
Number of clinical staff	Total number of clinical staff per 10 000 population	DHIMS2 for the year 2021	No transformation
Health Expenditure	Total district health expenditure per capita	Health expenditure for the year 2020/2021	No transformation
Exogenous variables			
*Health system factors*			
Health facility type	Proportion of PHC facilities (includes health centres, clinics & CHPS) over all health facilities	DHIMS2 for the year 2021	No transformation
Health insurance coverage	The proportion of the population covered by health insurance	Ghana Population and Housing Census 2021	Squared
*Socioeconomic factors*			
Literacy level	The proportion of the population that is literate	Ghana Poverty Mapping Report, 2015	Squared
Gini coefficient	A measure of inequality in welfare distribution	Ghana Poverty Mapping Report, 2015	Square root
Poverty incidence	The proportion of the population living below the national poverty line	Ghana Population and Housing Census 2021	Square root
Urbanisation	The proportion of the population living in urban areas	Ghana Population and Housing Census 2021	Square root
*Demographic factors*			
Population density	Number of persons per square kilometre	Ghana Population and Housing Census 2021	Log

ANC, Antenatal care; BCG, Bacillus Calmette-Guérin; CHPS, Community-based Health Planning and Services; DHIMS2, District Health Information Management System-2; DPT, Diphtheria, Tetanus, and Pertussis; LLINs, Long-Lasting Insecticidal Nets; TB, tuberclosis.

Table 1 outlines the variables used in the SFA model, their definitions, sources and data transformation. The input variables used in the efficiency estimation represent different types of resources, including:

1. Total number of public health facilities.
2. Total number of clinical staff.
3. District health expenditure.

The inputs were carefully selected to reflect physical, human and financial resources available for PHC service delivery in the districts. While we acknowledge that these variables do not fully reflect available resources in the districts, our choice was limited by readily available data. The number of public health facilities (hospitals, polyclinics, health centres, clinics, maternity homes and CHPS compounds) reflects physical infrastructure or capital, a basic requirement for our health production function. The clinical staff includes nurses, doctors and laboratory technicians, among others, who represent labour in our production function. A detailed list of health personnel by district is provided in online supplemental file 1. The health expenditure variable represents district-level spending on recurrent items like staff compensation and procurement of health commodities necessary for healthcare delivery in the districts. Most of the output and input data were obtained from the DHIMS2 for 2021 per district. The district health expenditure data was extracted from the budget allocation report for the year 2020/2021.

The output variable was a composite coverage index (CCI), a summary measure of service coverage.^29^ The CCI was calculated as a weighted average of coverage across eight key PHC interventions, grouped along four stages of the continuum of care: family planning, maternal and newborn care, immunisation and treatment of sick children.^30^ These stages represent critical points in the delivery of essential health services, particularly for women and children. Originally developed to support global health monitoring and equity assessments in LMICs, the CCI enables meaningful cross-district comparisons and is well-suited for efficiency analysis in such settings.

We selected the CCI because it aligns with Ghana’s UHC goals and the services typically delivered through the district-level PHC system. These output indicators are a fair reflection of the Ghana district PHC service delivery system, which plays key roles in providing preventive and curative care at the frontline. While the CCI is a widely used and policy-relevant metric, it predominantly reflects maternal, newborn and child health (MNCH) services. It does not explicitly account for other essential PHC components such as non-communicable disease (NCD) care, adult health or mental health services. Despite these limitations, it remains a practical proxy for evaluating PHC coverage in contexts with limited comprehensive routine data. Data on the district-level coverage of the eight PHC interventions were obtained from the DHIMS2 for the year 2021. The CCI was weighted and computed as detailed below and summarised in table 1.


$$CCI=\frac{1}{5}\left(FP+\frac{ANC4+SBA}{2}+\frac{BCG+2\times DPT3+MSL}{4}+LLIN+TB\right)$$

where:

CCI - composite coverage index,

FP - family planning demand satisfied with modern methods,

ANC4 - completed four antenatal care visits,

SBA - skilled birth attendance,

BCG - BCG vaccine coverage,

DPT3 - DPT3 vaccine coverage,

LLIN - long-lasting insecticide-treated net coverage and

TB - completed treatment of tuberculosis.

We used various exogenous variables from the health system and socioeconomic factors (see table 1). We used suitable transformations for non-normally distributed variables after examining their distributions. The most suitable transformation for each variable was determined using the *gladder* command in Stata (table 1).

### Data management and analysis

Data were collated into an Excel spreadsheet, cleaned and exported to Stata version 17 (StataCorp, College Station, TX, USA) for all the descriptive and inferential analyses. The analytical approach consisted of two stages. First, SFA was used to determine the technical efficiency of the production units, in this case, the districts, allowing us to rank districts according to a technical efficiency score and to compute the average technical efficiency score across districts. Second, we evaluated the relationship between the estimated efficiency and district characteristics.^31^ At this stage, *tobit* regression of the exogenous variables against the predicted efficiency scores was undertaken across the districts. Descriptive statistics, such as mean, SD, range of inputs, outputs and exogenous variables, were computed.

SFA is a parametric econometric technique used to estimate production or cost frontiers and measure efficiency across decision-making units like hospitals, districts and countries.^32 33^ Unlike traditional regression models, SFA introduces a composed error term where one component captures statistical noise (random shocks or measurement error) and the other represents inefficiency. The basic model expresses output as a function of inputs multiplied by a stochastic term reflecting these two effects. SFA is widely applied in fields such as healthcare for benchmarking performance, as it provides both frontier estimates and unit-specific efficiency scores, making it a key tool for identifying and addressing performance gaps.

We estimated output-oriented efficiency, assuming that each district could increase their outputs using a fixed quantity of inputs. We estimated output-oriented efficiency per district using the Battese and Coelli model, assuming the Cobb-Douglas functional form of production frontier due to its simplicity.^33 34^ The SFA model was specified as below:

$ln\;(output{)}_{i}=ln(HF_{i})+ln(CS_{i})+ln(Expenditure_{i})+(V_{i}-\mu_{i})$

where:

Output: district-level CCI,

Inputs: total number of health facilities (HF), clinical staff (CS) and health expenditure in each district.

V_i_: Symmetric random error term, accounting for statistical noise.

µ_i_: Non-negative inefficiency term, representing a deviation from the frontier (assumed to follow a distribution such as half-normal, truncated normal or exponential).

We then predicted the efficiency scores across the districts. To avoid the problem of multicollinearity in estimating the production function, we examined the correlation among and between inputs and exogenous variables using Pearson’s correlation coefficient. Correlation coefficients above a cut-off of 0.8 were considered to be highly correlated.^35^ This was followed by backward selection to include only less correlated variables in the analyses of associations between exogenous variables and technical efficiency. An exogenous variable with a p-value of <0.05 was considered statistically significant. The results are presented as coefficients with a 95% confidence interval (CI).

### Patient and public involvement

Patients and/or the public were not involved in the design, or conduct, or reporting, or dissemination plans of this research. The reflexivity statement for this paper is linked as an online supplemental file 2.

## Results

The descriptive statistics of inputs, outputs and exogenous variables are presented in table 2. On average, the population density across the districts was 1070 per km^2^, ranging between 14 and 16 759. There are wide differences between the minimum and maximum values for all the input and output variables, signifying uneven distribution and utilisation of resources across the districts. Specifically, the number of health facilities ranged from 1 to 13, clinical staff from 5 to 236 and expenditure from 7 to 1484 per 10 000 population. The mean CCI was 74% (SD: 8.2), ranging between 52% and 98% across the districts (table 2).

**Table 2:** Descriptive statistics of data on inputs, outputs and exogenous factors across districts in Ghana

Variable	No of observations	Mean	SD	Minimum	Maximum
CCI (%)	181	74.37	8.19	51.55	97.99
Population	181	130 367	69 602.42	38 268	443 981
Health facilities (per capita)*	181	3.44	1.50	1.00	13.30
Clinical staff (per capita)*	181	27.56	20.29	5.09	236.04
Budget (per capita)†	181	144.72	122.53	6.74	1483.71
Proportion of PHC facilities (%)	181	35.12	13.67	8.00	99.00
Health insurance coverage (%)	181	71.01	12.27	40.50	94.70
Literacy level (%)	181	66.00	17.12	19.00	93.90
Gini coefficient (%)	175	38.78	6.15	27.20	64.00
Poverty incidence (%)	175	27.19	19.49	1.30	92.40
Urbanisation (%)	181	48.84	27.32	0.00	100.00
Population density	181	1070.19	2797.94	13.50	16 759.30

* Per 10 000 population.

† in Ghana cedi.

CCI, composite coverage inde; PHC, Primary healthcare.

From the first stage of the SFA, the mean predicted efficiency across all the districts was 86.8% (SD: 6.58), ranging between 65.2 and 99.2%. The predicted technical efficiency scores for each district are provided in online supplemental file 3. A total of 175 (63.6%) districts were included in the second stage analysis of the determinants of efficiency. Table 3 outlines the correlation results among and between the inputs and exogenous variables. None of the variables was highly correlated. We, therefore, included all of them in the analysis of the factors.

**Table 3:** Pearson correlation among inputs and exogenous variables

Variable	1	2	3	4	5	6	7	8	9	12
No of HFs	1.00									
No. of clinical staff	0.38	1.00								
Budget	0.16	0.27	1.00							
Proportion of PHC facilities	0.38	−0.10	−0.07	1.00						
Health insurance coverage	0.36	0.23	0.18	0.12	1.00					
Literacy level	−0.12	0.11	0.18	−0.52	−0.01	1.00				
Gini coefficient	0.28	0.20	0.09	0.19	0.46	−0.19	1.00			
Poverty incidence	0.28	0.03	−0.02	0.40	0.22	−0.68	0.34	1.00		
Urbanisation	−0.38	0.07	0.11	−0.63	−0.03	0.67	−0.18	−0.53	1.00	
Population density	−0.10	0.02	0.09	−0.36	−0.19	0.40	−0.20	−0.36	0.59	1.00

HF, health facilities; PHC, Primary healthcare.

Findings from the second stage analysis revealed two factors significantly associated with technical efficiency within the districts. These were the proportions of PHC facilities and the Gini coefficient. The proportion of PHC facilities within a district was positively associated with the technical efficiency of the district PHC systems. Conversely, the Gini coefficient was negatively associated with efficiency. There was no statistically significant association between efficiency and health insurance coverage, literacy levels, urbanisation, population density or poverty incidence (table 4).

**Table 4:** Associations of exogenous variables with technical efficiency using a two-step SFA approach (n=175)

Exogenous variables	Regression coefficient	P value	95% CI
Proportion of PHC facilities	0.151	0.007*	0.041 to 0.261
Health insurance coverage	−0.038	0.067	−0.079 to 0.003
Literacy level	−0.002	0.955	−0.055 to 0.052
Urbanisation	0.022	0.275	−0.012 to 0.062
Population density	0.002	0.628	−0.010 to 0.006
Poverty incidence	−0.001	0.690	−0.006 to 0.004
Gini coefficient	−0.858	0.006*	−1.146 to −0.252

* statistically significant at a p-value of <0.05.

PHC, primary healthcare.

## Discussion

In this study, we have reported how efficiently PHC services are delivered at the district level in Ghana. The results suggest that while many districts perform reasonably well, there is still significant room to improve resource use. On average, districts could improve their efficiency in PHC service delivery by 13% without additional resources. However, the performance varies widely across the country. For example, 24 districts had efficiency scores below 80%, with some as low as 65%. This means they could potentially increase their PHC service output by 20% to 35% using the same inputs. On the other hand, only nine districts had efficiency scores above 95%. These findings highlight that Ghana’s district PHC systems are characterised by some technical inefficiencies. This is slightly lower than the level of inefficiency of an average of 30% reported from Kenya^36^ and 20% reported across the African Region.^37 38^

The second stage of regression of the efficiency scores against the exogenous variables revealed that a higher proportion of PHC facilities was associated with greater technical efficiency of the district PHC systems. In contrast, a higher Gini coefficient was associated with lower efficiency.

The positive association between a high proportion of PHC facilities and higher technical efficiency might be explained by the fact that a higher proportion of PHC facilities increases access to healthcare. In Ghana, the mean proportion of PHC facilities is 89%, with the highly efficient districts having a range of between 83% and 94%, while the least efficient districts have a range of between 63% and 87%. The positive association might be due to the CHPS initiative implemented in Ghana, which includes the construction of CHPS compounds and the deployment of community health officers and community health volunteers who deliver health services at the doorsteps of the community. The CHPS initiative has been reported to have improved access to and coverage of health services, including antenatal care and skilled deliveries.^39^,^46^

The Gini coefficient was negatively associated with the technical efficiency of the district PHC systems, signifying that higher income inequalities lead to inefficiency. This finding aligns with other research showing that when wealth is unequally distributed, fewer people can access and benefit from healthcare services.^22^ Interestingly, we did not find a significant association between efficiency and other factors like insurance coverage, education levels, poverty rates, urbanisation or population density. This contrasts with other studies that reported a significant association between efficiency and population density, urbanisation and literacy levels.^22 36^ The lack of association with health insurance coverage is of particular interest as it has close linkages with healthcare utilisation and delivery. The lack of significant association in Ghana may be attributed to the numerous implementation challenges facing the national health insurance scheme, reducing its effectiveness in improving healthcare service delivery.^10 47 48^

The current discussions call for relevant policies to ensure that health sector resources are used efficiently, particularly at the primary care level.^49 50^ The study’s findings highlight the need for district health departments to identify ways of improving the efficiency of district PHC systems. For instance, districts with a low proportion of PHC facilities could prioritise increasing their numbers while enhancing the readiness and availability of services in new and existing facilities. To ensure sustainable gains, strategies such as strengthening community health programmes by improving the availability of trained personnel, essential supplies and supportive supervision can enhance service delivery at the frontline. Moreover, optimising resource allocation and strengthening coordination mechanisms between district health management teams and facility leadership could mitigate inefficiencies in budget execution and workforce distribution. There is also a need for the national government, together with all relevant stakeholders, to scale up social protection programmes and policies to reduce poverty and inequality, which undermine the ability to seek healthcare services. Addressing structural issues such as income inequality, identified in our study as significantly associated with inefficiency, requires multisectoral action, including fiscal redistribution, education and employment programmes. Finally, implementing regular district-level efficiency audits and incorporating efficiency metrics into district performance frameworks may help institutionalise accountability and foster continuous improvement.

This study faced some limitations. A key limitation of this study was the use of a service coverage index, which predominantly captures MNCH and infectious disease services, excluding much of the broader PHC scope. This narrow focus does not capture the growing burden of NCDs, which now account for nearly half of all mortality in Ghana. Hence, the efficiency scores largely reflect MNCH performance rather than the full scope of PHC. Future research should expand coverage indicators to include a broader range of PHC services, including NCD care, for a more comprehensive efficiency assessment. Also, there was a lack of data for some output indicators, which may have impacted our estimates. Additionally, in estimating the technical efficiency of the district PHC system, we may not have fully accounted for various structural and organisational factors that influence service coverage that are challenging to quantify, such as political influence, facility locations and supervision capacity. These limitations underscore the importance of mixed methods approaches in evaluating efficiency, allowing for documentation of influential factors that may not be captured quantitatively. Finally, the study included data from only a year (2021), limiting our ability to analyse efficiency trends or technological changes over time. Future studies could benefit from covering additional years to better assess these trends.

## Conclusion

This study found that while PHC systems at the district level in Ghana are performing relatively well, there is still room to improve how efficiently resources are used. These differences suggest that some districts can deliver services more efficiently than others, though the underlying drivers may be complex and context specific. To enhance PHC performance in Ghana, focused efforts are needed to help less efficient districts to catch up. Policymakers aiming to strengthen the efficiency of PHC service provision in Ghana may wish to explore the factors associated with higher efficiency, such as increasing the proportion of PHC facilities and addressing within-district economic inequality. Strengthening efficiency across all districts could contribute to a fair and impactful use of health resources.

## Supplementary material

10.1136/bmjgh-2024-018847 online supplemental file 1

10.1136/bmjgh-2024-018847 online supplemental file 2

10.1136/bmjgh-2024-018847 online supplemental file 3

## Data Availability

Data are available upon reasonable request.
