# Supplementary material for: Level and determinants of district primary healthcare system technical efficiency in Ghana: two-stage stochastic frontier analysis
Source: BMJ Glob Health. 2026 Feb 12;11(2):e018847. doi: 10.1136/bmjgh-2024-018847 (PMC12911674; doi:10.1136/bmjgh-2024-018847)
Supplement: online supplemental file 2 [file bmjgh-11-2-s002.pdf]

**Appendix S1 – Author Reflexivity Statement**

**Title: Level and determinants of district primary health care system technical efficiency in Ghana: two-stage stochastic frontier analysis**

| Domain | Question | Response |
|---|---|---|
| Study conceptualisation | How does this study address local research and policy priorities? | This paper reporting the levels and determinants of district primary health care system technical efficiency addresses Ghana's UHC goals and PHC priorities as outlined in national policy documents. |
| | How were local researchers involved in study design? | Local researchers (BA, JN, RNDKM and EB) led the conceptualisation and design of the study, ensuring relevance to Ghana's health system and priorities. |
| Research management | How has funding been used to support the local research team(s)? | Funding supported fieldwork, analysis, and capacity building of the Ghanaian and Kenyan research team. |
| Data acquisition and analysis | How are research staff who conducted data collection acknowledged? | Data collection staff and collaborators were acknowledged in the authorship. |
| | How have members of the research partnership been provided with access to study data? | All research team members had shared access to cleaned datasets. |
| | How were data used to develop analytical skills within the partnership? | JN, FB and EB guided and mentored BA during data analysis and manuscript development. |
| Data interpretation | How have research partners collaborated in interpreting study data? | Monthly meetings were held jointly with partners in Kenya, Ghana, and the UK to review the analysis process, examine preliminary findings, and collaboratively interpret the data. |
| Drafting and revising for intellectual content | How were research partners supported to develop writing skills? | Early-career team members (BA and RNDKM) were guided by senior researchers in the team through drafting and reviewing manuscript sections. |
| | How will research products be shared to address local needs? | The paper will be published in an open-access journal, ensuring that researchers and policy makers in Ghana and other regions can easily |

| Domain | Question | Response |
| --- | --- | --- |
| | | access and engage with the findings. Moreover, the findings were disseminated through stakeholder meetings. |
| Authorship | How is the leadership, contribution and ownership of this work by LMIC researchers recognised within the authorship? | BA led the study and is appropriately listed as the first author. The authorship team includes strong representation from researchers based in LMICs (BA, JN, RNDKM and EB), supported by collaborators from high-income countries (FB and AV). |
| | How have early career researchers across the partnership been included within the authorship team? | Early career researchers are listed as the first (BA) and second authors (RNDKM) and were engaged in each stage of research. |
| | How has gender balance been addressed within the authorship? | The authorship includes a balanced representation of female and male researchers. |
| Training | How has the project contributed to training of LMIC researchers? | The study provided training in data analysis and writing for LMIC team members. |
| Infrastructure | How has the project contributed to improvements in local infrastructure? | The study did not contribute to improvements in the local infrastructure. However, it enhanced local research capacity through data systems and collaboration, and the findings offer recommendations for strengthening the district primary health care system in Ghana. |
| Governance | What safeguarding procedures were used to protect local study participants and researchers? | Ethical approvals were obtained, and anonymised data protocols were followed. |
